# Stable aqueous dispersion of superparamagnetic iron oxide nanoparticles protected by charged chitosan derivatives

**DOI:** 10.1007/s11051-012-1372-9

**Published:** 2012-12-22

**Authors:** Agnieszka Szpak, Gabriela Kania, Tomasz Skórka, Waldemar Tokarz, Szczepan Zapotoczny, Maria Nowakowska

**Affiliations:** 1 Faculty of Chemistry, Jagiellonian University, Ingardena 3, 30-060 Krakow, Poland; 2 H. Niewodniczanski Institute of Nuclear Physics, Polish Academy of Sciences, Krakow, Poland; 3 Department of Solid State Physics, AGH University of Science and Technology, al. A. Mickiewicza 30, 30-059 Krakow, Poland

**Keywords:** Chitosan, Superparamagnetic nanoparticles, SPION, Layer-by-layer deposition, Magnetic resonance imaging, Relaxivity

## Abstract

This article presents the synthesis and characterization of biocompatible superparamagnetic iron oxide nanoparticles (SPIONs) coated with ultrathin layer of anionic derivative of chitosan. The water-based fabrication involved a two-step procedure. In the first step, the nanoparticles were obtained by co-precipitation of ferrous and ferric aqueous salt solutions with ammonia in the presence of cationic derivative of chitosan. In the second step, such prepared materials were subjected to adsorption of oppositely charged chitosan derivative which resulted in the preparation of negatively charged SPIONs. They were found to develop highly stable dispersion in water. The core size of the nanocoated SPIONs, determined using transmission electron microscopy, was measured to be slightly above 10 nm. The coated nanoparticles form aggregates with majority of them having hydrodynamic diameter below 100 nm, as measured by dynamic light scattering. Their composition and properties were studied using FTIR and thermogravimetric analyses. They exhibit magnetic properties typical for superparamagnetic material with a high saturation magnetization value of 123 ± 12 emu g^−1^ Fe. Very high value of the measured *r*_2_ relaxivity, 369 ± 3 mM^−1^ s^−1^, is conducive for the potential application of the obtained SPIONs as promising contrast agents in magnetic resonance imaging.

**Electronic supplementary material:**

The online version of this article (doi:10.1007/s11051-012-1372-9) contains supplementary material, which is available to authorized users.

## Introduction

Magnetic resonance imaging (MRI) is one of the noninvasive diagnostic techniques used in medicine to visualize internal structures that, however, needs injection of a contrast agent to enable efficient imaging of different tissues. Complexes of gadolinium (Gd) ions are widely used as such contrast agents, (Caravan et al. 1999; Geraldes and Laurent 2009), but they may be toxic leading, e.g., in the case of patients with reduced renal function to the rare disease, nephrogenic systemic fibrosis (NSF) (Thomsen 2011; Haemel et al. 2011). Therefore, the development of an alternative material which could replace the contrast agents based on gadolinium is highly imperative. Toward this purpose, superparamagnetic iron oxide nanoparticles (SPIONs) have become the central points of the interest within the research community, because they are non-toxic and biodegradable/bioresorbable (Yang et al. 2008; Liu et al. 2011; Bhattacharya et al. 2012). These SPIONs also may exhibit better magnetic properties than gadolinium complexes implying the use of lower doses of these contrasts necessary to maintain good quality of MRI images. However, it has to be mentioned that both materials influence different types of relaxation times. The Gd-based contrast agents increase *T*_1_ relaxivity and thus they are used for *T*_1_-weighted imaging with positive contrast changes. SPIONs predominantly shorten *T*_2_ relaxation time and thus provide negative contrast in *T*_2_-weighted images.

There are many techniques in use for the fabrication of SPIONs (Laurent et al. 2008; Teja and Koh 2009; Tartaj et al. 2003; Mohanpuria et al. 2008), but only a few of them can be useful for medical applications because of possible contamination resulting from the technological processes used in the other cases. Co-precipitation of ferrous and ferric ions in an aqueous environment is commonly used since it does not require any hazardous organic solvents, extreme conditions of pressure, or temperature. Also its simplicity makes visible development of easy scaling-up procedures. On the other hand, the obtained nanoparticles, if uncoated, have the tendency to aggregate. Thus, to stabilize them, various organic and inorganic materials have been used to form not only protective coatings but also to enhance biocompatibility of SPIONs (Laurent et al. 2008; Gupta and Gupta 2005; Amstad et al. 2011; Lecommandoux et al. 2005). Many contrast agents based on SPIONs, especially commercial ones, are coated with dextran, a natural polysaccharide (Geraldes and Laurent 2009; Laurent et al. 2008; Sosnovik et al. 2008). The obtained nanoparticles are often characterized by relatively large sizes and wide particle size distribution that are not optimal from biological point of view. SPIONs for biomedical applications (MRI) should have diameters in the range of 10–100 nm to ensure prolonged time of circulation in the blood stream and efficient distribution of SPIONs in tissues (Gupta and Gupta 2005). Moreover, the dextran-coated contrast agents usually consist of larger dextran beads each of them hosting numerous nanoparticles (Amstad et al. 2011). Such structures limit the magnetic interactions of the nanoparticles with the surrounding aqueous medium and, owing to the presence of large amounts of the polymer, reduce the magnetization of the whole contrast agent (Hong et al. 2008). Uncoated SPIONs may also leak from such beads resulting in their re-aggregation (Laurent et al. 2008). Similarly, dextran, owing to their weak hydrogen-bond-type (Jung 1995) interactions with SPIONs, can desorb from the nanoparticles’ surface.

Chitosan is a natural, biocompatible, and biodegradable polysaccharide obtained by deacetylation of chitin (Rinaudo 2006). Chitosan chain has, in addition to hydroxyl group, also amine functional groups that broaden the scope of possible modifications of this polymer and its applications. Moreover, chitosan chelates metal ions, (Yuan et al. 2008) enhancing the interactions between the polymer chains and the surfaces of SPIONs with coordination bonds (Wang et al. 2008; Hernández et al. 2009). Unfortunately, to achieve the solubility of that polymer in water, the protonation of its amine groups is necessary. This occurs in acidic pH, which is not acceptable in many biological systems.

Therefore, in this study, we propose to modify chitosan with the cationic groups (Cho et al. 2006) which are charged in a broad range of pH, and above all, make the application of this polymer possible during the co-precipitation of SPIONs in highly basic solution. The nanoparticles obtained in such procedure should be stable in water and possess positive charge on the surface. What is more, we can easily change and control the surface charge by adsorption of reversely charged polyelectrolytes, as the surface charge is also important for biocompatibility issues (Amstad et al. 2011; Kralj et al. 2012). As we have shown recently (Bulwan et al. 2009), the most effective is the use of the polyelectrolytes with the same main polymeric chain in the layer-by-layer (LbL) deposition procedure that was adopted in this study, too. Importantly, SPIONs for successful application in vivo have to resist adsorption of biomacromolecules like proteins. Our previous studies indicated also that the chitosan-based coating applied here exhibit antifouling, anticoagulant, and antibacterial properties (Bulwan et al. 2012). This article presents the water-based synthesis and characterization of biocompatible SPIONs coated with ultrathin layer of ionic derivatives of chitosan, which develop highly stable dispersion in water.

## Experimental part

### Materials

**Scheme 1:**
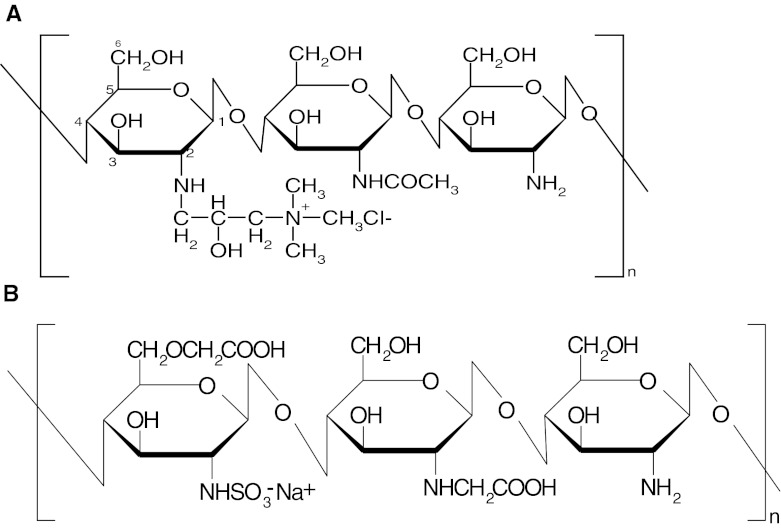
Structures of the chitosan derivatives **a** cationic derivative—CCh, **b** anionic derivative—ACh

Cationic (CCh) and anionic (ACh) derivatives of chitosan (low molecular weight, Sigma-Aldrich) (see Scheme 1) were synthesized and characterized according to the procedures described earlier (Bulwan et al. 2009). The degree of substitution of CCh by quaternary amine groups reached 95 ± 5 %, while for ACh, the percentage of the sulfonate groups was determined to be 66 ± 5 % with respect to the number of glucosamine units. Molecular weights of the polymers were determined using static light-scattering technique and found to be equal to 75 ± 13 kDa for CCh and 75 ± 6 kDa for ACh.

Iron(III) chloride hexahydrate (FeCl_3_·6H_2_O) and iron(II) chloride tetrahydrate (FeCl_2_·4H_2_O) as well as: ammonia (25 % solution, puriss. p.a.), methanol (purris. p.a.), acetone (purris. p.a.), and acetic acid (purris. p.a.) were obtained from Sigma-Aldrich. Mili-Q water was used in the preparation of the solutions.

### Synthesis of SPIONs

The synthesis of SPIONs was carried out in an aqueous solution. First, iron salts in molar ratio Fe(III):Fe(II) = 2:1, (0.1622 g FeCl_3_·6H_2_O and 0.0596 g FeCl_2_·4H_2_O), were dissolved in 50 mL of aqueous solution of CCh (*c* = 1 or 3 g L^−1^) obtaining the pH value of ca. 1.5. The solution was deoxygenated by purging with argon and sonicated (Sonic-6, Polsonic, 480 W, 1 s pulse per every 5 s) for 10 min in a thermostated bath at 20 °C. Afterward, 5 mL of 5 M NH_3(aq)_ was added dropwise, and the solution was further sonicated for 30 min. The obtained suspension was filtered by cellulose syringe filters (0.2 μm). Finally, the SPIONs were purified by magnetic chromatography. Toward this purpose, suspension of the SPIONs was injected into the column filled with ferromagnetic wires and exposed to high magnetic field. Such loaded column was flushed with water. Then, the magnets were removed, and the purified SPIONs were eluted from the column. SPION1 were obtained at the CCh concentration equal to 1 g L^−1^ and SPION3 at the concentration 3 g L^−1^, respectively.

To obtain the negatively charged SPIONs, the synthesized nanoparticles were additionally coated with anionic derivative of chitosan (ACh) using LbL method. Aqueous solution of ACh (10 mL, *c* = 1 g L^−1^ in 0.1 M NaCl) was added to the SPION1 suspension (4 mL) and sonicated continuously for 10 min at 25 °C. The coated nanoparticles, SPION1/ACh, were purified using magnetic chromatography, as described above.

### Methods

The average molecular weights of chitosan derivatives were determined using static light-scattering technique on Zetasizer Nano ZS instrument (Malvern) equipped with He–Ne laser operating at 633 nm. The measurements were performed at least for three different concentrations of the polymers in acetic buffer (pH = 3.7) at 25 °C. Toluene (HPLC grade) was used as a standard RI.

The size and the size distribution of the nanoparticles were characterized by TEM [Tecnai G2 F20 (200 kV) with field emission gun]. The bright-field and high-resolution electron microscopy (HREM) images were obtained. The aqueous dispersions of SPIONs were sonicated for 2 min before deposition on a carbon film support and air-dried at room temperature. For crystallographic measurements, GATAN digital micrograph was used applying fast Fourier transformation (FFT) analysis. The size of the SPIONs was determined from TEM images taking into account at least 100 measurements for each sample. Such formed histograms were fitted with Gaussian curves, and the averaged diameter values were given as the ones in the maximum of the fitted curves with the respective standard deviations.

Hydrodynamic sizes and zeta potentials of the nanoparticles were measured at constant ionic strength equal to 0.01 M using dynamic light scattering (DLS, ZetaSizer Nano ZS) at 173° scattering angle. The measurements were performed at 25 °C. Standard algorithms implemented in the software provided by the manufacturer were applied to obtain size distribution of particles by intensity and by number.

Thermogravimetric analyses were performed using Netzsch STA 449 F1 Jupiter in the temperature range of 23–810 °C in argon flow and using platinum crucible. The heating rate was set to 10 °C min^−1^.

A classical spectrophotometric method based on absorbance measurements of the color complex of iron(II) with phenanthroline was used for determination of iron content in the SPIONs. Toward this purpose, 1 mL of the SPIONs dispersion was dissolved in 1 mL of hot 1 M HCl. Then, an excess of vitamin C (with respect to the anticipated iron content) was introduced for reducing Fe(III) to Fe(II). Finally, 3 mL of 0.2 % solution of phenanthroline in 0.08 M HCl was added to the obtained solution. The sample was left in dark for 10 min, and its absorbance at *λ* = 512 nm (characteristic for the formed complex) was measured. Based on the previously prepared calibration, the concentration of iron in the sample was determined.

FTIR spectra were collected using a Varian 620-IR system with a liquid cooled mercury–cadmium–telluride (MCT) detector. The samples of both bare and coated nanoparticles were measured in transreflection mode. The samples were prepared by deposition of the colloidal suspension on a dedicated MirrIR glass (Kevley Technologies) and left overnight for evaporation of water. CCh powder was measured using attenuated total reflection (ATR) mode with a diamond ATR crystal.

The measurements of magnetization were performed for suspension as well as dry samples using Lake Shore 7300 vibrating sample magnetometer and constant-flow cryostat (Janis). AC (frequency *ν* = 189 Hz) magnetic susceptibility values for the suspensions of SPIONs were measured in a capsule (Lake Shore Cryotronic Inc., 700 SC-10) at 295–323 K temperature range. Stanford SR 830 lock-in nanovoltmeter was used for controlling the current.

The ^1^H *T*_1_ and *T*_2_ relaxation times were measured using the 200.11 MHz MRI system consisting of MARAN-DRX spectrometer (Resonance Instruments. Ltd.), horizontal 4.7 T/310 mm superconducting magnet (Bruker), and the home-built imaging probe-head with inductively coupled birdcage radio-frequency coil of the diameter equal to 40 mm. The probe was mounted on the animal bed and positioned in the center of the magnet using the spoiled-GRE imaging sequence. The probe shimming and the pulse-length adjustments were made before each experiment. The inversion recovery (IR) and Carr–Purcell–Meiboom–Gill (CPMG) sequences were used to measure *T*_1_ and *T*_2_, respectively; in the CPMG experiments, 128–2,048 echoes were acquired with the echo times ranging from the shortest available 200–400 μs. Fourteen data points were measured in the IR experiment. They were uniformly distributed on the logarithmic time scale in the 10 ms–12 s range. The repetition time was equal to 12 s. No accumulation was used in either IR or CPMG acquisition. Single-exponential curves were obtained and fitted in the time domain using the specialized spectrometer procedures.

The effect of SPIONs on erythocytes present in human blood was investigated through visual observation using optical microscopy (ECLIPSE LV100D, Nikon). A droplet of blood (4 μL) was mixed with 18 μL of SPIONs dispersion (various concentrations) on a glass plate and analyzed in comparison to the sample of untreated blood.

## Results and discussion

### Synthesis of SPIONs

The negatively charged SPIONs with biocompatible thin polymer coating were synthesized in a two-step procedure. In the first step, the iron oxide nanoparticles were formed from a mixture of FeCl_3_ and FeCl_2_ salts by reacting with ammonia in the presence of cationic derivative of chitosan (CCh). Chitosan was intentionally modified with charged quaternary amine groups to achieve its solubility in the basic solution of the reaction mixture upon addition of ammonia. The role of this polymer was to complex the iron ions and thus to limit the growth of the nanoparticles during co-precipitation. When polyanions, such as poly(acrylic acid), are used for this purpose, they rather form gels with iron(III) ions (Yokoi et al. 1993). The parameters of the SPIONs synthesis were carefully optimized varying the conditions, such as concentration of the reagents, temperature, stirring method and speed, and sonication protocol, to obtain the nanoparticles of preferred properties. Sonication was applied in all experiments because it was found to be more effective in the avoidance of nanoparticle aggregation than mechanical or magnetic stirring. Owing to the positive correlation observed between the reaction temperature and the size, as well as the size distribution of SPIONs, the reaction medium was cooled and thermostated at 20 °C. Also, the appropriate sonication protocol was applied to prevent the rapid heating of the sample usually occurring during continuous sonication. Magnetic chromatography applied here enabled efficient and easy purification of SPIONs from the remaining reagents. The presence of the cationic polymer, CCh, during the co-precipitation leads to fabrication of stable aqueous dispersion of small aggregates of SPIONs required for biomedical applications. This can be explained considering the fact that SPIONs formed in a presence of CCh exhibited highly positive zeta potential—greater than 40 mV for both polymer concentrations used during the synthesis (see Table 1). There is practically no precipitation in the suspension of SPIONs after a few weeks' storage time. For longer times, some aggregation can be noticed; however, good dispersion may be easily recovered by applying the short sonications. While the other parameters of SPIONs also did not significantly differ for both concentrations of CCh used in the synthesis, only SPION1 were chosen for further modifications.

**Table 1:** Zeta potential values for SPION dispersions obtained under various conditions

Sample	Zeta potential (mV)
SPION1 (*c*_CCh_ = 1 g L^−1^)	+47 ± 9
SPION3 (*c*_CCh_ = 3 g L^−1^)	+42 ± 5
SPION1/ACh	−41 ± 6

Such positively charged nanoparticles obtained, in the second step of the fabrication process, were coated with ACh leading to SPION1/ACh. The LbL technique based on the alternating adsorption of oppositely charged polyelectrolytes was employed here. Thanks to high positive surface charge of SPION1 covered by CCh and the same backbone of the oppositely charged ACh, the adsorption was very efficient as indicated by inversion of the zeta potential to the value reaching even less than −40 mV for SPION1/ACh (see Table 1). This value also ensures the stability of the aqueous dispersion of those coated SPIONs.

The efficiencies of the synthetic procedures were determined based on the iron content in the final, purified suspensions. The theoretical content of iron was calculated based on the feed composition of the reaction mixture. The efficiencies of 24 % for SPION1 and 39.9 % for SPION3 reflected rather, not the level of conversion, but the loss of material in the form of larger aggregates during the purification procedure (filtering and magnetic chromatography).

### Size and size distribution of SPIONs

**Fig. 1:**
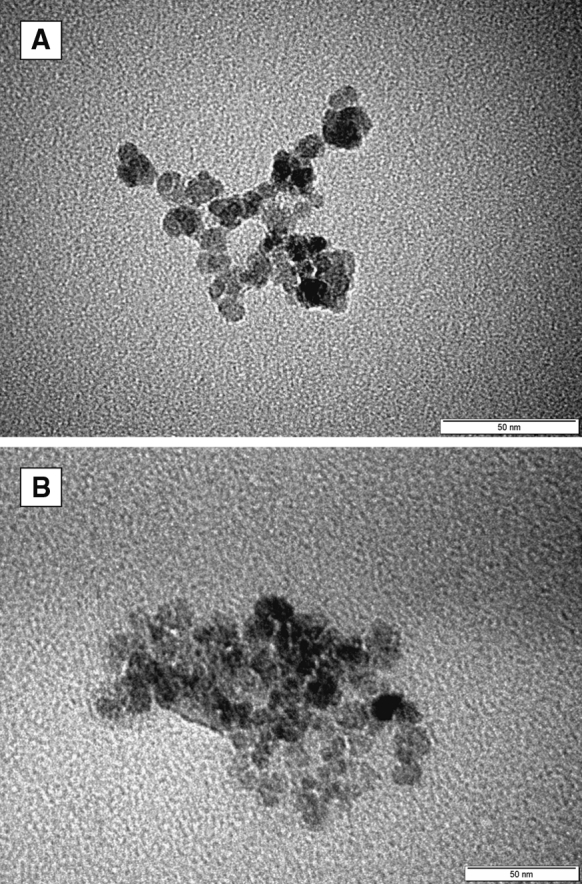
Bright-field TEM image for SPION1 (**a**) and SPION1/ACh (**b**)

**Fig. 2:**
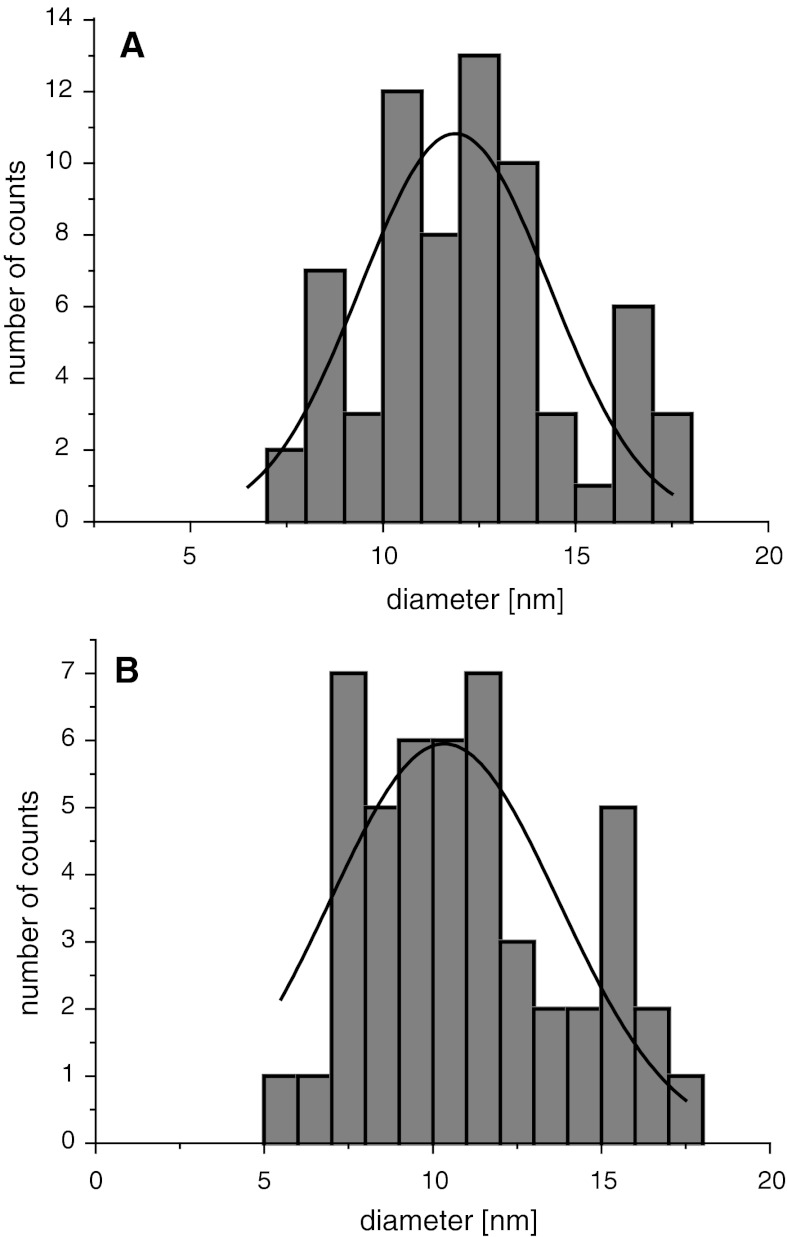
Distribution of the diameters SPION1 (**a**) and SPION1/ACh (**b**) as determined from TEM images

TEM was applied to measure the shape and size of the synthesized nanoparticles (see Fig. 1). The image shows well-separated nanoparticles within a small aggregate. The core sizes of SPION1 were found to be very small with an average diameter being equal to 11.9 ± 1.7 nm (see Fig. 2). The size of the nanoparticles upon coating with ACh remained practically the same (*d* = 10.1 ± 2.9; Fig. 2). This can be explained considering that ACh layer is ultrathin, which is typical for LbL films formed as a result of electrostatic self-assembly of polyelectrolytes used (Crouzier et al. 2010), and it does not provide sufficiently efficient contrast in TEM. Relatively low standard deviation values indicate narrow distributions of the nanoparticle diameters as preferred for biological applications.

**Fig. 3:**
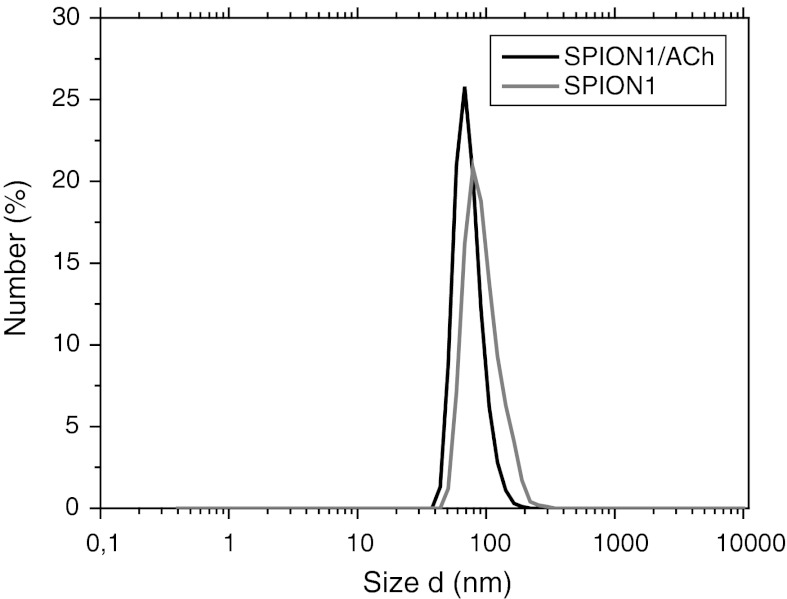
DLS results of hydrodynamic diameter distribution by number for SPION1 and SPION1/ACh

DLS measurements have shown that in aqueous dispersion, nanoparticles exist in the form of small aggregates. The data indicated that hydrodynamic diameters of the obtained SPION1 and SPION1/ACh aggregates are generally less than 100 nm (see Fig. 3). The average diameter for negatively charged SPION1/ACh seems to be slightly smaller than the size of the precursor SPION1. This can be ascribed to some differences in the thicknesses of the electric double layers for both the studied samples (e.g., slightly different zeta potential), which contribute to the total hydrodynamic size measured by DLS. Moreover, the measured diameters may be also affected by surface-bound polymeric chains which slow the diffusion and increase the apparent size of the particles/aggregates. Nevertheless, DLS results are not able provide a direct support for successful coating of the SPION1 since the determination of the diameters of these particles can be significantly affected by other parameters, including also the dynamic character of the formed aggregates. The obtained values are reasonable taking into account the hydration of the chitosan coated and highly charged nanoparticles. The coating is evidenced by measurements of zeta potential. Moreover, it has been reported that relaxivity values (see later for details) of such clusters are greatly improved when compared to the values for isolated nanoparticles. That is essential for MRI applications (Berret et al. 2006; Matsumoto and Jasanoff 2008).

### Physicochemical characterization of SPIONs

**Fig. 4:**
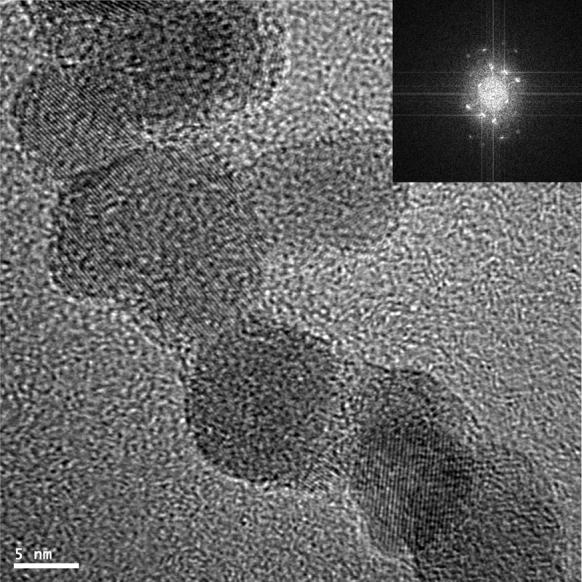
HREM image of SPION1. FFT analysis of the image is in the *inset*

HREM images for SPION1 (see Fig. 4) were also used for determination of the crystal structure of the obtained nanoparticles. It appears that the nanoparticles are mostly composed of single domains, which also implies their preferred magnetic properties (see later). The interplanar crystal spacings were measured and compared with crystal spacings for the iron (II, III) oxides (see Table 2).

**Table 2:** Crystallographic parameters determined for SPION1 from HREM images and those for Fe_3_O_4_ (Iidaa et al. 2007) and γ-Fe_2_O_3_ (Lesin et al. 2010) taken from the literature

Measured interplanar crystal spacing for SPION1 (Å)	Interplanar crystal spacing for Fe_3_O_4_ (Å)	Interplanar crystal spacing for Fe_2_O_3_ (Å)	Miller’s indicators
4.82	4.85	4.81	111
3.06	2.97	2.97	220
2.58	2.53	2.52	311
2.80	2.81	–	221
1.68	1.71	1.70	422
1.55	1.56	–	423

It has been reported that under the anaerobic conditions applied in the co-precipitation method, Fe_3_O_4_ is preferentially formed (Gupta and Gupta 2005). However, the crystallographic structures of Fe_3_O_4_ (magnetite) and Fe_2_O_3_ (maghemite) are very similar, and it is very difficult to confirm the presence of exclusively one phase in the synthesized SPIONs. Nevertheless, one can conclude that the presence of CCh during the precipitation does not negatively affect the structures of SPIONs and their magnetic properties (see later) leading to the formation of highly crystalline iron oxide nanoparticles.

**Fig. 5:**
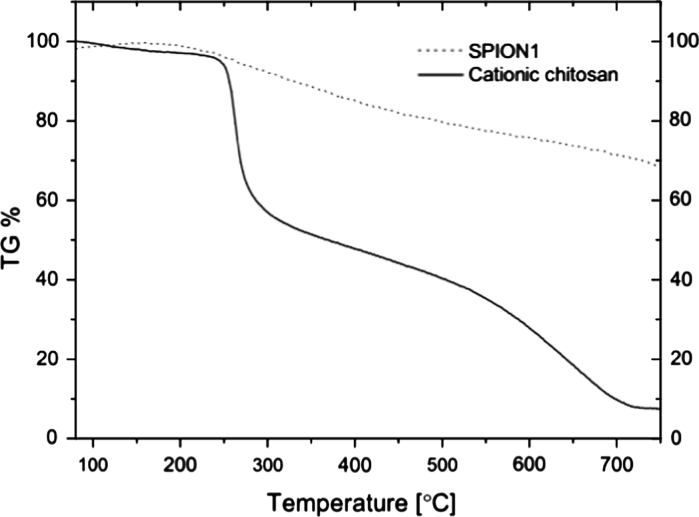
TGA analysis for CCh and SPION1 containing CCh

To confirm the presence of chitosan layers and to determine their content in the nanoparticles, thermogravimetric analysis (TGA) was performed. Figure 5 shows TGA curves for SPION1 and for CCh as the only component of the nanoparticles that decompose in the applied temperature range. The observed behavior is similar to the one reported for ferrogels containing chitosan (Hernández et al. 2009). Based on the TGA data, it was found that the content of CCh in SPION1 sample was around 32 %. That seems to be a reasonable value that is necessary to densely coat the nanoparticles, while it is not too high to significantly decrease the magnetization of this hybrid material (see later).

**Fig. 6:**
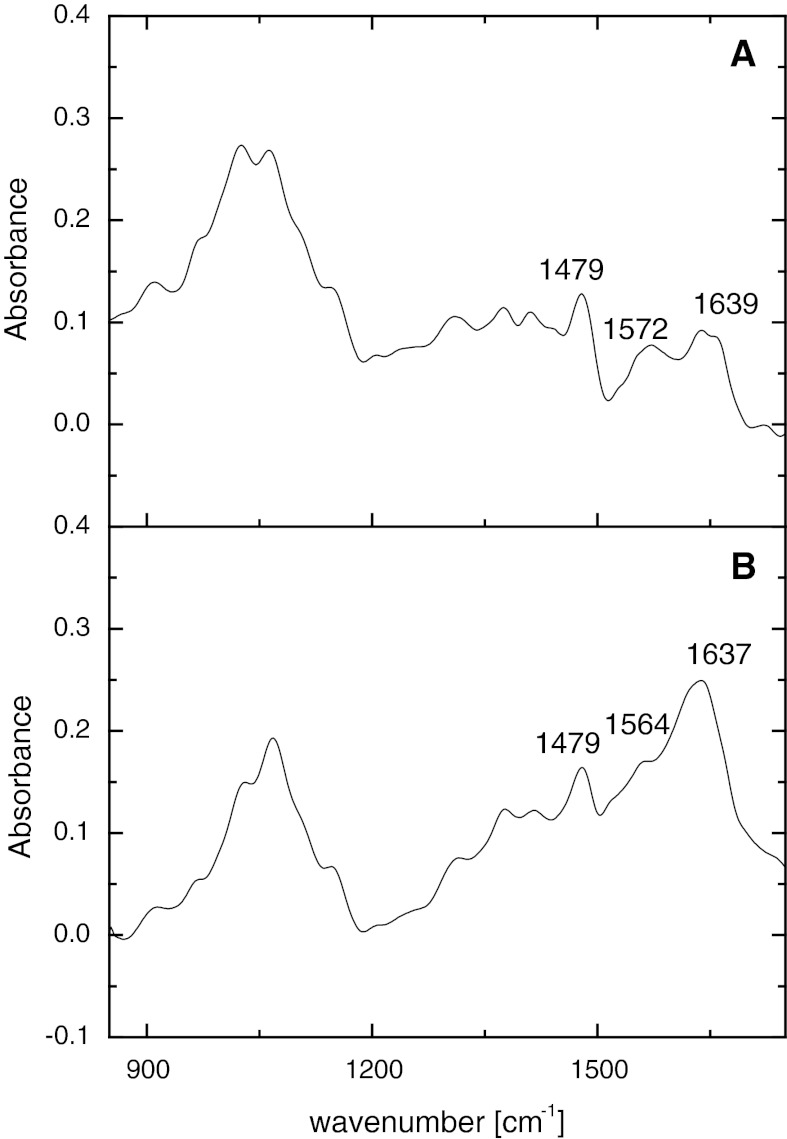
FTIR spectra of CCh (**a**) and SPION1 (**b**)

The interactions between CCh polymer and iron in the hybrid SPION1 were later studied using FTIR (see Fig. 6). There are characteristic peaks at 1,479 cm^−1^ for the methyl band of GTMAC, at 1,572 cm^−1^ for the primary amine group (Cho et al. 2006) and at 1,639 cm^−1^ for the amide I band (Wang et al. 2008; Hernández et al. 2009) in the spectrum of CCh. The similarity of the overall shape of the FTIR spectra for SPION1 and CCh confirm again the presence of CCh in the hybrid nanoparticles. Moreover, the shift of the peak at 1,564 cm^−1^ indicates the interaction of the primary amine group with the iron ions and the creation of amino complexes (Hernández et al. 2009) as postulated above.

### Magnetic properties of SPIONs

**Fig. 7:**
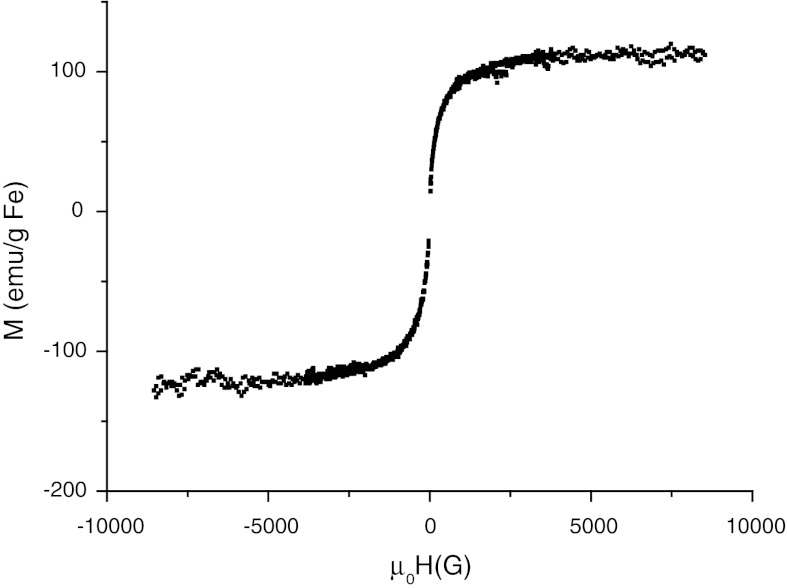
The magnetization of the nanoparticle suspension in a function of magnetic field for SPION1 (at 200 K)

The magnetic properties of the fabricated SPIONs were evaluated using various methods. The magnetization in a function of magnetic field was measured for SPION1. The shape of the curve in Fig. 7 and the absence of the hysteresis confirm the superparamagnetic properties of the obtained SPIONs. The results ( Fig. 7) indicate that the nanoparticles are highly magnetic, with the saturation magnetization being equal to 123 ± 12 emu g^−1^ Fe—very close to the value measured for magnetite (Jung and Jacobs 1995). Taking into account the total mass of the coated nanoparticles, the saturation magnetization reaches the value of 61 ± 6 emu g^−1^, which is comparable to the ones measured for similar superparamagnetic nanoparticles (Matsumoto and Jasanoff 2008). Magnetic susceptibility of SPION1 was also determined in 295–323 K temperature range. The real part of susceptibility was found to be ca. 1.4 cm^3^ g^−1^ and slightly decreasing for higher temperatures. Positive value of this parameter is characteristic for paramagnetic material. The FC/ZFC (field cooling/zero field cooling) measurements were also performed for dried SPION1 (see Electronic Supplementary Materials). However, the obtained results suggest further aggregation of SPION1 after drying and cannot be directly compared to the particles’ diameter measured for nanoparticle suspension (see Fig. 3).

### Relaxometry of SPIONs

In order to determine relaxivity *r*_1_ and *r*_2_ for SPIONs, the following relations were used:

1 $$ R_{1} = \frac{1}{{T_{1} }}\quad R_{2} = \frac{1}{{T_{2} }} $$

2 $$ R_{1} = R_{1}^{0} + r_{1} \cdot c_{\text{Fe}} \quad R_{2} = R_{2}^{0} + r_{2} \cdot c_{\text{Fe}} $$

where *R*_1_ (*R*_2_) is the longitudinal (transverse) relaxation rate; *T*_1_ (*T*_2_) is the longitudinal (transverse) relaxation time; *R*_1_^0^ (*R*_2_^0^) is the longitudinal (transverse) relaxation rate in the absence of SPIONs; *r*_1_ (*r*_2_) is the longitudinal (transverse) relaxivity; and *c*_Fe_ is the iron concentration in the samples.

**Fig. 8:**
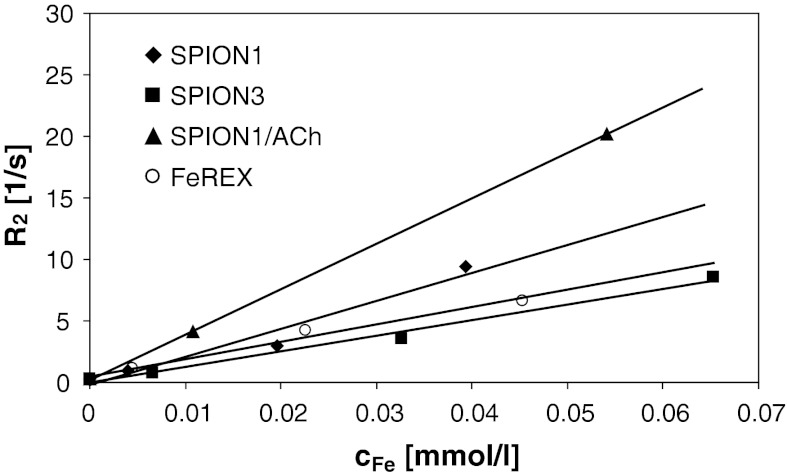
Dependence of transverse relaxation rate on concentration of iron in magnetic nanoparticles

Values of relaxivity, *r*_1_ and *r*_2_, indicate whether SPIONs could be effective contrast agents for MRI. The iron oxide nanoparticles are contrast agents affecting *T*_2_; so for the best performance, *r*_2_ values should be very high, while *T*_1_ values are only weakly dependent on the contrast concentration implying low *r*_1_ values. As presented in Fig. 8 and Table 3, the fabricated SPIONs are characterized by relatively high *r*_2_ values that are also comparable to the values determined under the same measurement conditions for the commercial contrast agent, FeREX. It should be noticed that for negatively charged nanoparticles, SPION1/ACh, *r*_2_ was determined to be especially high and equal to 369 ± 3 mM^−1^ s^−1^ which is larger by more than 2.5 times than the value for FeREX. These materials seem to be very attractive for MRI applications, considering also that other commercial contrast agents like Sinerem/Combidex or Endorem/Feridex have *r*_2_ values equal to 65 and 120 mM^−1^ s^−1^, respectively (both determined at 1.5 T) (Laurent et al. 2008).

**Table 3:** Relaxivity values for dispersions of the obtained SPIONs and the commercial MRI contrast agent (FeREX)

Sample	*r*_1_ (mM^−1^ s^−1^)	*r*_2_ (mM^−1^ s^−1^)
SPION1	1.99 ± 0.45	227 ± 36
SPION3	1.72 ± 0.19	128 ± 10
SPION1/ACh	1.56 ± 0.19	369 ± 3
FeREX	1.93 ± 0.16	141 ± 13

The observed relatively high relaxivity values for the obtained nanoparticles may be explained in terms of enhancement effect of the highly hydrated coating that has been only recently postulated (de Haan and Paquet 2011). For the highly charged polymeric coatings applied in this study, one can expect high hydration, definitely much higher than that functioning for uncharged or even hydrophobic coatings. It has to be stressed that water diffusion in the presence of the particles with the hydrophilic polymer shells may be hampered, what has been proven by observation of decrease of the water diffusion coefficients measured for that kind of polymers (Kwak and Lafleur 2003). This is evidenced here by significant increase of *r*_2_ value (see Table 3) only after deposition of ACh on SPION1 without any change in the inorganic part of the nanoparticles and with only small variation in the average size of the aggregates.

## Conclusion

Biocompatible superparamagnetic iron oxide nanoparticles (SPIONs) coated with ultrathin layer of ACh were prepared in a simple water-based two-step procedure. The core size of the nanocoated SPIONs was slightly above 10 nm, while hydrodynamic diameter of the hydrated both negatively and positively charged aggregates of nanoparticles in aqueous dispersion was in the range 80–100 nm (maxima of the size distributions). Owing to the electrostatic repulsions between these particles, they form stable dispersion in aqueous medium. The materials display the preferred magnetic properties with very high values of saturation magnetization (123 ± 12 emu g^−1^ Fe) and transverse relaxivity (369 ± 3 mM^−1^ s^−1^), suggesting their potential application as contrast agents in MRI. This indicates that although nanoparticles form the aggregates in aqueous dispersion, they preserve their excellent magnetic properties, which suggests that within the aggregates, the individual nanodomains of iron oxide coated with chitosan layer still exist. This approach based on proper design of ultrathin coatings on superparamagnetic nanoparticles may pave the way for the preparation of high-performance MRI contrast agents. Moreover, the applied coatings enable tailoring charge and surface chemistry for specific biomedical applications. Biological trials which aim at testing this hypothesis are currently in progress.

## Electronic supplementary material

Below is the link to the electronic supplementary material.

Supplementary material 1 (DOC 114 kb)

## References

[CR1] Amstad E, Textor M, Reimhult E (2011). Stabilization and functionalization of iron oxide nanoparticles for biomedical applications. Nanoscale.

[CR2] Berret JF, Schonbeck N, Gazeau F, El Kharrat D, Sandre O, Vacher A, Airiau M (2006). Controlled clustering of superparamagnetic nanoparticles using block copolymers: design of new contrast agents for magnetic resonance imaging. J Am Chem Soc.

[CR3] Bhattacharya D, Sahu SK, Banerjee I, Das M, Mishra D, Maiti TK, Pramanik P (2012). Synthesis, characterization, and in vitro biological evaluation of highly stable diversely functionalized superparamagnetic iron oxide nanoparticles. J Nanopart Res.

[CR4] Bulwan M, Zapotoczny S, Nowakowska M (2009). Robust one-component chitosan-based ultrathin films fabricated using layer-by-layer technique. Soft Matter.

[CR5] Bulwan M, Wójcik K, Zapotoczny S, Nowakowska M (2012). Chitosan-based ultrathin films as antifouling, anticoagulant and antibacterial protective coatings. J Biomater Sci Polym Ed.

[CR6] Caravan P, Ellison JJ, McMurry TJ, Lauffer RB (1999). Gadolinium(III) chelates as MRI contrast agents: structure, dynamics, and applications. Chem Rev.

[CR7] Cho J, Grant J, Piquette-Miller M, Allen C (2006). Synthesis and physicochemical and dynamic mechanical properties of a water-soluble chitosan derivative as a biomaterial. Biomacromolecules.

[CR8] Crouzier T, Boudou T, Picart C (2010). Polysaccharide-based polyelectrolyte multilayers. Curr Opin Colloid Interface Sci.

[CR9] de Haan HW, Paquet C (2011). Enhancement and degradation of the R2* relaxation rate resulting from the encapsulation of magnetic particles with hydrophilic coatings. Magn Reson Med.

[CR10] Geraldes CFGC, Laurent S (2009). Classification and basic properties of contrast agents for magnetic resonance imaging. Contrast Media Mol Imaging.

[CR11] Gupta AK, Gupta M (2005). Synthesis and surface engineering of iron oxide nanoparticles for biomedical applications. Biomaterials.

[CR12] Haemel AK, Sadowski EA, Shafer MM, Djamali A (2011). Update on nephrogenic systemic fibrosis: are we making progress?. Int J Dermatol.

[CR13] Hernández R, Zamora-Mora V, Sipaja-Ballestero M, Vega-Baudrit J, López D, Mijangos C (2009). Influence of iron oxide nanoparticles on the rheological properties of hybrid chitosan ferrogels. J Colloid Interface Sci.

[CR14] Hong RY, Feng B, Chen LL, Liu GH, Li HZ, Zheng Y, Wei DG (2008). Synthesis, characterization and MRI application of dextran-coated Fe_3_O_4_ magnetic nanoparticles. Biochem Eng J.

[CR15] Iidaa H, Takayanagia K, Nakanishib T, Osakaa T (2007). Synthesis of Fe_3_O_4_ nanoparticles with various sizes and magnetic properties by controlled hydrolysis. J Colloid Interface Sci.

[CR16] Jung CW (1995). Surface properties of superparamagnetic iron oxide MR contrast agents: ferumoxides, ferumoxtran, ferumoxsil. Magn Reson Imaging.

[CR17] Jung CW, Jacobs P (1995). Physical and chemical properties of superparamagnetic iron oxide MR contrast agents: ferumoxides, ferumoxtran, ferumoxsil. Magn Reson Imaging.

[CR18] Kralj S, Rojnik M, Romih R, Jagodic M, Kos J, Makovec D (2012). Effect of surface charge on the cellular uptake of fluorescent magnetic nanoparticles. J Nanopart Res.

[CR19] Kwak S, Lafleur M (2003). NMR self-diffusion measurements of molecular and macromolecular species in dextran solutions and gels. Macromolecules.

[CR20] Laurent S, Forge D, Port M, Roch A, Robic C, Elst LV, Muller RN (2008). Magnetic iron oxide nanoparticles: synthesis, stabilization, vectorization, physicochemical characterizations, and biological applications. Chem Rev.

[CR21] Lecommandoux S, Sandre O, Chécot F, Rodriguez-Hernandez J, Perzynski R (2005). Magnetic nanocomposite micelles and vesicles. Adv Mater.

[CR22] Lesin VI, Koksharov YA, Khomutov GB (2010). Magnetic nanoparticles in petroleum. Petrol Chem.

[CR23] Liu F, Laurent S, Fattahi H, Elst LV, Muller RN (2011). Superparamagnetic nanosystems based on iron oxide nanoparticles for biomedical imaging. Nanomedicine.

[CR24] Matsumoto Y, Jasanoff A (2008). T2 relaxation induced by clusters of superparamagnetic nanoparticles: Monte Carlo simulations. Magn Reson Imaging.

[CR25] Mohanpuria P, Rana NK, Yadav SK (2008). Biosynthesis of nanoparticles: technological concepts and future applications. J Nanopart Res.

[CR26] Rinaudo M (2006). Chitin and chitosan: properties and applications. Prog Polym Sci.

[CR27] Sosnovik DE, Nahrendorf M, Weissleder R (2008). Magnetic nanoparticles for MR imaging: agents, techniques and cardiovascular applications. Basic Res Cardiol.

[CR28] Tartaj P, del Morales Puerto M, Veintemillas-Verdaguer S, González-Carreño T, Serna CJ (2003). The preparation of magnetic nanoparticles for applications in biomedicine. J Phys D Appl Phys.

[CR29] Teja AS, Koh PY (2009). Synthesis, properties, and applications of magnetic iron oxide nanoparticles. Prog Cryst Growth Charact Mater.

[CR30] Thomsen HS (2011). Contrast media safety—an update. Eur J Radiol.

[CR31] Wang Y, Li B, Zhou Y, Jia D (2008). Chitosan-induced synthesis of magnetite nanoparticles via iron ions assembly. Polym Adv Technol.

[CR32] Yang J, Gunn J, Dave SR, Zhang M, Wang YA, Gao X (2008). Ultrasensitive detection and molecular imaging with magnetic nanoparticles. Analyst.

[CR33] Yokoi H, Nomoto E, Ikoma S (1993). Reversible formation of iron(III) ion clusters in the poly(acrylic acid)–Fe_3_+ complex gel with changes in the water content. J Mater Chem.

[CR34] Yuan Q, Venkatasubramanian R, Hein S, Misra RDK (2008). A stimulus-responsive magnetic nanoparticle drug carrier: magnetite encapsulated by chitosan-grafted-copolymer. Acta Biomater.

