# Supplementary material for: Stable aqueous dispersion of superparamagnetic iron oxide nanoparticles protected by charged chitosan derivatives
Source: J Nanopart Res. 2012 Dec 22;15(1):1372. doi: 10.1007/s11051-012-1372-9 (PMC3568472; doi:10.1007/s11051-012-1372-9)
Supplement: Supplementary file 1 — Supplementary material 1 (DOC 114 kb) [file 11051_2012_1372_MOESM1_ESM.doc]

**Electronic Supplementary Materials**

**Stable Aqueous Dispersion of Superparamagnetic Iron Oxide Nanoparticles**

**Protected by Charged Chitosan Derivatives**

**Agnieszka Szpak,^a^ Gabriela Kania,^a^ Tomasz Skórka,^b^ Waldemar Tokarz,^c^ Szczepan Zapotoczny,*^a^ Maria Nowakowska*^a^**

*^a^ Jagiellonian University, Faculty of Chemistry, Ingardena 3, 30-060 Krakow, Poland, phone: +48 12 6632250, fax: +48 12 6340515*

*^b^ H. Niewodniczanski Institute of Nuclear Physics, Polish Academy of Sciences, Krakow, Poland*

*^c^AGH University of Science and Technology, Department of Solid State Physics, al. A. Mickiewicza 30, 30-059, Krakow, Poland.*

*email: zapotocz@chemia.uj.edu.pl, nowakows@chemia.uj.edu.pl*

1. FC/ZFC measurements.

Fig. 1S. FC/ZFC measurements for dried SPION1 in function of temperature.

The blocking temperature, T_B_, was calculated from ZFC by fitting a quadratic function and found to be 190±30 K. Based on those results the diameter of the formed aggregates (after dying) was estimated to be 1.1±0.6 μm (Gitleman et all 1974). The value is much higher than the one obtained for suspension but it is clear that during evaporation concentration of the nanoparticels increases and it implies further aggregation.

Gittleman J I, Abeles B, Bozowski S (1974) Superparamagnetism and relaxation effects in granular Ni-SiO_2_ and Ni-Al_2_O_3_ films. Rev B 9:3891-3897
